# Relationships between Problematic Cannabis Use and Risky Behaviors in Spanish Adolescents

**DOI:** 10.3390/ijerph16173029

**Published:** 2019-08-21

**Authors:** Raquel Alarcó-Rosales, Miriam Sánchez-SanSegundo, Rosario Ferrer-Cascales, Natalia Albaladejo-Blázquez, Nicolás Ruiz-Robledillo, Elisa Delvecchio, Javier Oltra-Cucarella

**Affiliations:** 1 Department of Health Psychology, Faculty of Health Science, University of Alicante, 03690 Alicante, Spain; 2 Department of Philosophy, Social Sciences and Education; Università degli Studi di Perugia, 06123 Perugia, Italy

**Keywords:** cannabis use, risky behaviors, Spanish adolescents, weekly available money, motor vehicle accident, physical assault

## Abstract

This study examined the relations between problematic cannabis use, physical assault, and getting involved in a motor vehicle accident under the influence of cannabis in a sample of adolescents randomly selected from 25 public and semiprivate high schools in Alicante (Spain). Participants (*n* = 648) completed The Spanish National Standardized Survey about drug use in high school adolescents (ESTUDES, 2017), which includes the cannabis abuse screening test (CAST). Prevalence of cannabis use across the life-span and within the past 30 days was 37.5% and 17.4%, respectively. CAST scores were associated with an increased risk of driving under the effects of cannabis, riding shotgun, and physical assault, but not with an increased risk of having a motor vehicle accident. There were no differences between boys and girls in the association of problematic cannabis use with risky behaviors. This result highlights the importance of comprehensive prevention and education strategies for adolescents at high risk of cannabis use.

## 1. Introduction

Adolescence is a critical developmental period for initiation of substance use and other risky behaviors. Experimentation of substance use often occurs during this time, increasing rapidly from early to late adolescence [1]. Early onset of substance use has been associated with an increased risk for adverse consequences among adolescents, including physical and mental health problems, brain abnormalities, school dropout, and educational underachievement [2,3,4,5,6]. Epidemiological studies have demonstrated that alcohol, tobacco, and cannabis are the most frequently used substances among adolescents resulting in negative health status in adulthood [7,8,9]. Further, there is accumulated evidence showing a pattern of progression of drug abuse from alcohol and tobacco to cannabis and other illicit drugs [6]. For example, Hingson et al. [3] found that individuals who began drinking before age 14 years were more likely to experience alcohol dependence ever and after 10 years of first drinking, and they were more likely to experience multiple dependence episodes compared to those who began drinking after the age 21 years. Macleod et al. [10] found an association between cannabis use and educational attainment, psychological health problems, problematic behaviors, and greater use of other drugs in adulthood. In addition, those who started using cannabis during adolescence were more likely to show a wide range of persistent cognitive deficits even after long abstinence periods [11]. 

Recent studies have highlighted the potential association between cannabis use prior to age 18 and physical assaults, motor vehicle collisions, and suicide [12,13]. Driving under the influence of cannabis has been associated with an increased risk of injuries and mortality [14,15]. For example, in a sample of 907 children from New Zealand, Fergusson, Swain-Campbell, and Horwood [15] found a relationship between cannabis abuse and car accidents. These findings are consistent with the results by Schwartz, Hoffmann, and Jones [16], who reported that cannabis-dependent adolescents were at risk of suicide and getting involved in a motor vehicle accident under the influence of marijuana.

Although most findings reported on cannabis-related consequences among adolescents have been found to be consistent across cultures, most of the current literature on cannabis use comes from North America and some European countries, and while some findings may be consistent across countries, cannabis abuse may be perceived differently across cultural context. In Spain, according to the National Standardized Survey about drug use [17], around 20% of adolescents aged 14 to 18 years reported cannabis use at least once during their lives, with the estimated prevalence of last-year cannabis use being 17.1% for young adults aged 15–34 years. According to the report on victims of motor vehicle accidents during 2017 in Spain published by the National Institute of Toxicology [18], 884 people died because of a motor vehicle accident. Of these, 58.25% were positive on cannabis tests, either in isolation or in combination with other drugs. Among those testing positive on drug tests after death because of motor vehicle accidents, 32 (6.21%) were less than 25 years old. 

These data must be highlighted, because cannabis use in Spanish adolescents has been associated with high-risk consumption of cocaine, ecstasy, amphetamines, and hallucinogens, with no significant differences between boys and girls [19]. However, to our knowledge, there have been no previous studies in Spanish adolescents on the association of cannabis use with the risk of motor vehicle accidents and other risky social behaviors. Understanding the association between problematic cannabis use and potential risky behaviors for mortality is crucial to the development of effective strategies for prevention. This work aimed to analyze the relations between problematic cannabis use and getting involved in motor vehicle accidents and physical assault under the influence of cannabis, after controlling for demographic variables. 

## 2. Materials and Methods 

### 2.1. Sample

The present study was a part of a large-scale cross-sectional study on drug abuse and risky behaviors conducted in schools in Alicante, Spain. The study was approved by the University of Alicante, the Generalitat Valenciana for Education, Research, Culture and Sport, the Ayuntamiento de Alicante (AYTOALICANTE3-18I) for the National Drugs Plan and by the Educational Directive Committee from Schools involved in the study (UA2015-1013). Potential participants were high school students from 14 to 20 years randomly selected from 25 public and semiprivate high schools in Alicante. Per the Spanish educational law, public high schools are fully funded by the Spanish government, whereas semiprivate high schools are partially funded by the Spanish government and by other sources. 

Inclusion criteria were: (a) presence in the classroom on the day of the survey, (b) ability to read and complete the questionnaires themselves, and (c) being fluent in Spanish. Prior to conducting the study, the board of each participating school provided the students’ parents or legal guardian for children under 18 years with written information about objectives, methods, and evaluation processes, in accordance with the Spanish Royal Decree 1720/2007 on the Protection of Personal Data in children. All participants and parents or legal guardian were required to provide a written informed consent, explaining that their authorization might be revoked or cancelled at any time according to their own will. Students who were present the day of data collection and consented to participate in the study were instructed to complete an anonymous online survey in school. All participants received accurate, understandable, and appropriate instructions about how to answer the survey and were assured of the confidentiality of their responses. Participants were retained in the final sample only if they responded to all the questions analyzed in the present study. Data were collected in the classroom in presence of a research assistant from the University of Alicante during the second and third trimester of the 2018/2019 academic year, and sessions lasted approximately 60 min.

### 2.2. Assessment Procedure 

Participants completed the Spanish National Survey of Drug Abuse in high school students (ESTUDES) [17], a Spanish national standardized survey about drug use in high school adolescents. ESTUDES includes several modules, from which relevant information can be obtained regarding sociodemographic details, leisure activities, drug use (e.g., tobacco, alcohol, cannabis, cocaine), the influence of others, and health and social problems derived from drug use. ESTUDES was administered individually and anonymously. 

Demographic variables included in this work were age, sex, weekly money amount available, and type of high school (public vs. semiprivate). To analyze problematic cannabis use, the ESTUDES questionnaire includes six items that form the cannabis abuse screening test (CAST), a short tool to screen for cannabis use disorders [20]. The CAST has shown high internal consistency [20] and has been proven to be sensitive to screening for cannabis use disorders and dependence [21]. 

The CAST assesses the frequency of six events within the past 12 months related to harmful cannabis use: nonrecreational use (“Have you smoked cannabis before midday?”; “Have you smoked cannabis when you were alone?”), memory disorders (“Have you had memory problems when you smoked cannabis?”), being encouraged to reduce or stop using cannabis (“Have friends or family members told you that you should reduce or stop your cannabis use?”), unsuccessful attempts to quit (“Have you tried to reduce or stop your cannabis use without succeeding?”), and problems linked to cannabis use (“Have you had problems because of your cannabis use (argument, fight, accident, poor results at school, etc.)?”). Every item is answered using a 5-point Likert type scale (o = never, 1 = rarely, 2 = from time to time, 3 = quite often, 4 = very often), which provides a total score ranging from 0 to 24. Scores higher than 6 and 11 indicate moderate and severe addiction in a Spanish population of adolescents and young adults [22].

To analyze risky behaviors linked to problematic cannabis use, participants answered whether they had experienced within the past 12 months any of the following events: (i) driven a motor vehicle under the effects of cannabis; (ii) ridden shotgun with someone driving under the effects of cannabis; (iii) been involved in a motor vehicle accident within two hours after cannabis use; or (iv) been involved in a fight, or committed a physical assault, within two hours after cannabis use. Each question was answered with YES or NO responses. The answers to each of these four questions were the main outcome variables in the present study.

### 2.3. Statistical Analysis

Prevalence of cannabis use was calculated as the proportion of participants reporting having used cannabis at least once ever and at least once in the last month. Cannabis use included any cannabidiol (CBD)-containing product (e.g., marijuana, smoked hash, hash cookies, CBD oil, bongs). To calculate the amount of cannabis consumption, those who reported having used cannabis during the last month were requested to indicate the number of joints smoked per day. Nonparametric Mann–Whitney tests were used for continuous variables because of non-normal distributions. Categorical variables were analyzed with Chi^2^ tests. The association between problematic cannabis use and risky behaviors was assessed with a series of logistic regressions, with each of the four questions regarding risky behaviors as a dependent variable separately. In each regression, demographic variables and CAST total score were introduced as predictors in a block. To analyze whether the association between problematic cannabis use and risky behavior differed for males and females, we included a CAST x Sex interaction in the adjusted regression models. Statistical significance was set at 0.05 with Bonferroni correction in each block of comparisons. 

## 3. Results

During the assessment period, 885 students were approached. Of these, 17 participants refused to participate, and 37 participants did not attend school the assessment day. Eight hundred and thirty-one students completed the ESTUDES questionnaire. Data from 648 participants were analyzed for this study after excluding 183 participants with missing data in the ESTUDES questionnaire. As previous research has shown that multiple imputation did not change the results [23], missing data were not computed.

Means and frequencies for demographic variables are shown in Table 1. For four comparisons, there were no statistically significant differences in weekly available money between males and females (z = −0.20, *p*_corrected_ > 0.999). Males (Mean = 16.03, SD = 0.97) were slightly older than females (Mean = 15.75, SD = 0.87), with this difference being statistically significant (z = −3.59, *p*_corrected_ = 0.001). Participants in public schools (Mean = 16.04, SD = 0.97) were slightly older than participants in semiprivate schools (Mean = 15.56, SD = 0.73), with this difference being statistically significant (z = −6.07, *p*_corrected_ < 0.001), but had a similar amount of weekly available money (z = −1.98, *p*_corrected_ = 0.188). 

Two hundred and forty-three (37.5%) participants reported cannabis use at least once during their lifetime, and 113 (17.4%) reported cannabis use within the last month. Among participants reporting cannabis use within the last month, 43 (38.05%) smoked marijuana joints, 22 (19.47%) smoked hash joints, 42 (37.17%) smoked both, and 6 (5.31%) smoked electronic cigarettes, with an average consumption of 3.5 (SD = 3.53, range: 1–20) joints per day. Twenty-four (21.24%) participants reported using bongs, and 8 (7.08%) reported using cannabis cookies. Eighty-nine (78.76%) participants reported using a single cannabis delivery method. The average age of onset of cannabis use was 14.55 (SD = 1.19, range: 12–18) years.

Mean CAST scores (Table 2) were not indicative of cannabis use disorders. For two comparisons (Bonferroni corrected *p* ≤ 0.025), there were no statistically significant differences in CAST scores between males and females (z= −0.09, *p*_corrected_ > 0.999) or between participants in public or semiprivate schools (z = −0.71, *p*_corrected_ = 0.952). According to the cutoff points for the CAST, 4% and 2.6% of the participants scored in the moderate addiction and severe addiction range, respectively. More than 90% reported that they had never or rarely had behaviors related to cannabis use within the past 12 months, with percentages similar for males and females in each of the CAST items. 

The frequencies of risky behaviors are shown in Table 3. The probability of getting involved in risky situations for health and well-being after cannabis use was 0.3% (motor vehicle accident) to 6.7% (riding shotgun) for males and 1.2% (motor vehicle accident) to 7.5% (riding shotgun) for females. 

Logistic regression analyses for each risky behavior showed that CAST scores were associated with the risk of driving under the effects of cannabis (unadjusted OR = 1.20, SE = 0.305, *p* < 0.001), riding shotgun (unadjusted OR = 1.36, SE = 0.214, *p* < 0.001), and being involved in a fight (unadjusted OR = 1.34, SE = 0.039, *p* < 0.001). The association between CAST scores and the risk of driving a motor vehicle under the effects of cannabis became nonsignificant after including covariates in the regression models (Table 4). As only five participants reported having had a motor vehicle accident after cannabis use, its association with other variables could not be calculated. 

We then repeated the logistic regressions shown in Table 4 to analyze the association between risky behaviors and the number of joints per day for participants who reported cannabis use within the last month. The number of joints was not statistically associated with driving under the effects of cannabis (unadjusted OR = 1.14, SE = 0.068, *p* = 0.053), riding shotgun (unadjusted OR = 1.05, SE = 0.057, *p* = 0.393), or being involved in a fight (unadjusted OR = 0.78, SE = 0.593, *p* = 0.671). These results remained unchanged when covariates were included in the model. 

## 4. Discussion

The present work aimed to analyze the relations between cannabis use and risk behaviors that can have an impact on the quality of life and well-being in a sample of adolescents from the city of Alicante (Spain). Data from 648 participants were analyzed that included age, sex, weekly available money, the type of school, the frequency of cannabis use, and the frequency of risky behaviors such as driving a motor vehicle after cannabis use, riding shotgun with someone driving under the effects of cannabis, having been involved in a motor vehicle accident within two hours after cannabis use, and having been involved in a fight or a physical assault within two hours after cannabis use. Our results showed that 37.5% and 17.4% of the adolescents reported cannabis use ever and within 30 days, respectively, with no differences between males and females either in cannabis use or in risky behaviors after cannabis use. 

Our results show a prevalence of cannabis use during lifetime and within 30 days similar to that reported by other groups in different countries. Peters, Bae, Barrington-Trimis, Jarvis, and Leventhal [23] reported on a prevalence of cannabis use during lifetime and within 30 days of 33.9% and 14.9%, respectively, in a sample of 3177 adolescents from Los Angeles (USA) with a mean age of 16 years. Sampasa-Kanyinga, Hamilton, LeBlanc, and Chaput [24] reported on a prevalence of 21.5% for cannabis use within 12 months and a prevalence of 13.9% for cannabis use within 30 days in a sample of 9920 adolescents from Ontario, Canada. Results from adolescents in Spain showed an estimated prevalence of last-year cannabis use of 17.1% in young adults aged 15–34 years [17]. Indeed, it has been suggested that the pattern of substance use in Spain has changed among adolescents, showing a higher prevalence of cannabis plus tobacco (12.7%) use in comparison to tobacco (10.5%) or cannabis (2.1%) use alone [19]. Using the CAST cutoff points obtained from a sample of Spanish adolescents and young adults [22], we found a prevalence of 4% for moderate cannabis addiction, similar to the 3.8% of adolescents using cannabis with high risk reported on by Rial et al. [19] using cutoff points obtained from a sample of French adolescents [25]. 

Cannabis use has been associated with antisocial problematic behaviors. In a review of 48 longitudinal studies on cannabis use, Macleod et al. [10] reported that some studies found an association between increased cannabis use and increased problems, whereas other studies found no associations. Similarly, some studies reported an association between cannabis use and antisocial or otherwise psychological problems, whereas others failed to find such an association or, when found, it was attenuated after adjustment for potential confounding factors. However, younger age was consistently associated with an increased prevalence of subsequent problems. In a sample of 3882 adolescents, Rial et al. [19] found that users of tobacco and cannabis had a higher risk of sexting, accessing websites with erotic content, and getting drunk. The results reported in this study are in line with previous works reporting an association between cannabis use and risk behaviors and add to the existing literature that cannabis users scoring higher in the CAST might be more likely to ride shotgun with someone under the effects of cannabis, or being involved in a fight under the effects of cannabis. Our results showed that the most frequent risky behavior in both males and females was riding shotgun, which could partially explain the low prevalence of motor vehicle accidents (0.77%). However, data on the association between cannabis use and motor vehicle accidents must be interpreted cautiously because of the low prevalence of motor vehicle accidents in our sample. CAST scores reflect problematic cannabis use, so it would be interesting to analyze whether recreational cannabis use is also associated with motor vehicle accidents, either or not under the effects of cannabis. Using only CAST scores, it will not be possible to capture the association between cannabis use and motor vehicle accidents in recreational cannabis users.

Contrary to previous studies [10], age was not associated with problematic cannabis use and risky behaviors. This could be due to the narrow age range in our sample in comparison with the studies reviewed by Macleod et al. [10] that included participants aged 5–10 years and participants until age 27 years. As car license in Spain can be obtained from age 18 on, it is possible that including participants older than 18 may provide a stronger association between cannabis use and risky behaviors, mainly those behaviors related to driving. In a study with 907 participants aged 21 from New Zealand, Fergusson et al. [15] found that the percentage of individuals reporting cannabis use increased with increasing self-reported risky driving behaviors, from 3.6% among those reporting no risky driving behaviors to 51.8% among those reporting seven or more risky driving behaviors from age 18–21 years. However, participants in our sample were requested to indicate whether they had been involved in a motor vehicle accident, including cars but also motorbikes. According to the Spanish law on driving license, motor vehicle accidents under age 18 years could only include motorbike accidents, as motorbike license can be obtained from age 14 on. Thus, the comparison with studies reporting car accidents might not be appropriate. 

The main limitation of the present study, as it occurs with previous reports [10,15], is the use of self-reports to analyze both the frequency of cannabis use and the frequency of risky behaviors. Additionally, other variables, such as psychological problems, have been associated with cannabis use, either as its cause or as its consequence [21], but that could not be identified because of a lack of psychiatric interviews. Another limitation is the use of corrected *p*-values, which, as happened in the work by Peters et al. [23], turned into nonsignificant some results that would have been significant at *p* < *0*.05, as used in previous works [19,24]. This is the case with the association between weekly available money and the probability of getting involved in physical assault. However, even if significant, the association found in our sample between the amount of weekly available money and getting involved in physical assault would be negligible. Additionally, the association between CAST scores and risky behaviors reported in the present work relates to problematic cannabis use, but not to safely cannabis use. Indeed, our results showed that when the number of joints was included as a measure of cannabis use, no significant association was found between cannabis use and risky behaviors. As pointed out by a reviewer, the results reported here would not apply to adolescents who smoke in the afternoon with friends and show no signs of problematic cannabis use.

Our analyses did not include other confounding factors associated with cannabis use, such as social environment [26] and school–class factors [27]. Our results are observational, and no causality can be inferred from this study, mostly because cannabis use can increase in social environments where risk perceptions or substance-use norms are lower or where dissatisfaction with school exists [27], which could increase the likelihood of cannabis use in the first place. Thus, risk perception of cannabis and other drugs use might influence the relationship between cannabis use and risky behaviors and should be included in future studies. 

## 5. Conclusions

The results of the present study show that the prevalence of cannabis use among adolescents from the city of Alicante (Spain) is similar to the prevalence from other countries, with no differences in prevalence between males and females. However, the probability of getting involved in risky situations increased as the frequency of problematic cannabis use increased, with a similar risk between males and females of getting involved in the assessed risky behaviors. The main recommendation is to integrate the ESTUDES questionnaire in both public and private schools to identify adolescent cannabis users at a greater risk of risky behaviors, such as physical assault. Futures studies will analyze the value of tools such as the CAST questionnaire to predict risky behaviors prospectively. 

## Figures and Tables

**Table 1:** Means (standard deviations) and frequencies of the main study variables (*n* = 648).

Variables	Mean (SD)	Range	% (95%CI)
Sex (female)			334 (51.5%)
Public school			442 (68.2%)
Age	15.87 (0.93)	(14.41–19.28)	
Weekly available money (in euros)	13.25 (15.02)	(0–100)	
CAST total score	0.97 (2.90)	(0–20)	
CAST score = 0 (no problematic cannabis use) (*n* = 543)			83.8% (80.8–86.4)
CAST score = 1–6 (mild problematic cannabis use) (*n* = 62)			9.6% (7.5–12.1)
CAST score = 7–11 (moderate cannabis addiction) (*n* = 26)			4.0% (2.7–5.8)
CAST score ≥ 12 (severe addiction) (*n* = 17)			2.6% (1.6–4.2)

CAST: cannabis abuse screening test.

**Table 2:** Frequency of problems associated to cannabis use according to the cannabis abuse screening test.

CAST		Male		Female			
		Number	%	Number	%	*Chi^2^*	*p*_corrected_
“Have you smoked cannabis before midday?”	Never	276	87.9%	286	85.6%		
	Rarely	15	4.8%	16	4.8%		
	From time to time	18	5.7%	16	4.8%		
	Quite often	1	0.3%	7	2.1%		
	Very often	4	1.3%	9	2.7%	6.14	>0.999
“Have you smoked cannabis when you were alone?”	Never	280	89.2%	303	90.7%		
	Rarely	17	5.4%	4	1.2%		
	From time to time	7	2.2%	8	2.4%		
	Quite often	5	1.6%	8	2.4%		
	Very often	5	1.6%	11	3.3%	11.36	0.138
“Have you had memory problems when you smoked cannabis?”	Never	301	95.9%	306	91.6%		
	Rarely	7	2.2%	18	5.4%		
	From time to time	3	1.0%	7	2.1%		
	Quite often	1	0.3%	2	0.6%		
	Very often	2	0.6%	1	0.3%	6.54	0.972
“Have friends or family members told you that you should reduce or stop your cannabis use?”	Never	292	93.0%	314	94.0%		
	Rarely	8	2.5%	5	1.5%		
	From time to time	6	1.9%	9	2.7%		
	Quite often	4	1.3%	2	0.6%		
	Very often	4	1.3%	4	1.2%	2.14	>0.999
“Have you tried to reduce or stop your cannabis use without succeeding?”	Never	297	94.6%	313	93.7%		
	Rarely	7	2.2%	8	2.4%		
	From time to time	3	1.0%	8	2.4%		
	Quite often	4	1.3%	3	0.9%		
	Very often	3	1.0%	2	0.6%	2.49	>0.999
“Have you had problems because of your cannabis use (argument, fight, accident, poor results at school, etc.)?”	Never	299	95.2%	316	94.6%		
	Rarely	6	1.9%	10	3.0%		
	From time to time	5	1.6%	4	1.2%		
	Quite often	2	0.6%	1	0.3%		
	Very often	2	0.6%	3	0.9%	1.49	>0.999

**Table 3:** Frequency of risky behaviors related to cannabis use for males and females.

Risky Behaviors	Male (*n* = 314)		Female (*n* = 334)	
	Number	% (95% CI)	Number	%
Driving a motor vehicle under the effects of cannabis	11	3.5% (1.9–6.2)	6	1.8% (0.8–3.9)
Riding shotgun with someone driving under the effects of cannabis	21	6.7% (4.4–10)	25	7.5% (5.1–10.8)
Having a motor vehicle accident within two hours after cannabis use	1	0.3% (0.1–1.8)	4	1.2% (0.5–3)
Being involved in a fight, or committing physical assault, within two hours after cannabis use	14	4.5% (2.7–7.3)	14	4.2% (2.5–6.9)

**Table 4:** Logistic regressions for risky behaviors.

Risky Behaviors	Variable	OR	95% CI	*p*_corrected_
Driving a motor vehicle under the effects of cannabis	Age	1.04	0.62–1.75	>0.999
	Sex	1.62	0.45–5.82	>0.999
	Money available	1.01	0.98–1.04	>0.999
	School	2.79	0.61–12.79	>0.999
	CAST score	1.15	1.00–1.32	0.282
	CAST*Sex	1.08	0.89–1.29	>0.999
Riding shotgun with someone driving under the effects of cannabis	Age	1.12	0.78–1.61	>0.999
	Sex	0.82	0.34–1.95	>0.999
	Money available	1.01	0.99–1.03	>0.999
	School	1.43	0.63–3.24	>0.999
	CAST score	1.31	1.19–1.44	<0.001
	CAST*Sex	1.06	0.91–1.23	>0.999
Being involved in a fight, or committing physical assault, within two hours after cannabis use	Age	1.09	0.69–1.72	>0.999
	Sex	1.32	0.41–4.27	>0.999
	Money available	1.02	1.00–1.04	0.236
	School	0.81	0.31–2.13	>0.999
	CAST score	1.32	1.19–1.47	<0.001
	CAST*Sex	1.01	0.86–1.19	>0.999

CAST: cannabis abuse screening test. OR: odds ratio. CI: confidence interval.
